# Strengthening the Bolivian pharmacovigilance system: New surveillance strategies to improve care for Chagas disease and tuberculosis

**DOI:** 10.1371/journal.pntd.0008370

**Published:** 2020-09-21

**Authors:** Nuria Cortes-Serra, Ruth Saravia, Rosse Mary Grágeda, Amílcar Apaza, Jorge Armando González, Brenda Ríos, Joaquim Gascón, Faustino Torrico, María-Jesús Pinazo

**Affiliations:** 1 ISGlobal, Hospital Clínic - Universitat de Barcelona, Barcelona, Spain; 2 Fundación CEADES, Cochabamba, Bolivia; 3 Programa Departamental de Chagas, Cochabamba, Bolivia; 4 Programa Departamental de Tuberculosis, Cochabamba, Bolivia; 5 Unidad de Medicamentos y Tecnología en Salud (UNIMED), Área de Farmacovigilancia, Bolivia; 6 Unidad de Medicamentos y Tecnología en Salud (UNIMED), Área de Vigilancia y Control, Bolivia; Center for Biologics Evaluation and Research, Food and Drug Administration, UNITED STATES

## Abstract

Chagas disease (CD) and tuberculosis (TB) are important health problems in Bolivia. Current treatments for both infections require a long period of time, and adverse drug reactions (ADRs) are frequent. This study aims to strengthen the Bolivian pharmacovigilance system, focusing on CD and TB. A situation analysis of pharmacovigilance in the Department of Cochabamba was performed. The use of a new local case report form (CRF) was implemented, together with the CRF established by the Unidad de Medicamentos y Tecnología en Salud (UNIMED), in several healthcare centers. Training and follow-up on drug safety monitoring and ADR reporting was provided to all health professionals involved in CD and TB treatment. A comparative analysis of the reported ADRs using the CRF provided by UNIMED, the new CRF proposal, and medical records, was also performed. Our results showed that out of all patients starting treatment for CD, 37.9% suffered ADRs according to the medical records, and 25.3% of them were classified as moderate/severe (MS). Only 47.4% of MS ADRs were reported to UNIMED. Regarding TB treatment, 9.9% of all patients suffered ADRs, 44% of them were classified as MS, and 75% of MS ADRs were reported to UNIMED. These findings show that the reinforcement of the Bolivian pharmacovigilance system is an ambitious project that should involve a long-term perspective and the engagement of national health workers and other stakeholders at all levels. Continuity and perseverance are essential to achieve a solid ADR reporting system, improving patient safety, drug efficacy and adherence to treatment.

## Author summary

Chagas disease and tuberculosis are important public health problems in Bolivia, where the prevalence and incidence of these diseases are high compared to other Latin American countries. Treatment for both infections requires a long period of time, and adverse drug reactions (ADRs) are frequent. One of the main challenges in the management of Chagas disease and tuberculosis is poor adherence to treatment. In this context, strengthening the Bolivian pharmacovigilance system is proposed as a key issue to reinforce therapeutic strategies. Several interventions were carried out in fifteen health centers of the Department of Cochabamba and included a situation analysis of pharmacovigilance, the implementation of a new local case report form, specific training on drug safety monitoring and reporting, and follow-up visits. ADRs were also analyzed. Overall, our findings suggest that the Bolivian pharmacovigilance system still presents some challenges that should be addressed in the coming years in order to achieve a strong, integrated and consolidated ADR reporting system, which will be essential for avoiding patient abandonment and achieving therapeutic success.

## Introduction

Chagas disease (CD), caused by the parasite *Trypanosoma cruzi (T*.*cruzi)*, is one of the main health problems in Latin America. *T*. *cruzi* infection has been declared a major public health issue in the region, affecting approximately 6 to 7 million people worldwide. Bolivia is the country with the highest prevalence and incidence of CD. Additionally, travel and immigration patterns have increased the importance of *T*. *cruzi* infection outside of endemic areas [1–3].

CD is one of the 17 neglected tropical diseases (NTDs), which are characterized by affecting populations with poor socioeconomic status and having limited resources and political priority [1]. Approved drugs for treatment of *T*. *cruzi* infection (benznidazole and nifurtimox) are complex, and adverse events reactions (ADRs) are frequent. Onset of ADRs is one of the main causes of patients abandoning treatment, resulting in therapeutic failure or ineffective treatment [4–9].

Tuberculosis (TB) is another important health problem in Bolivia, where the prevalence and incidence of the disease is high compared to other South American countries. TB treatment involves the combination of several drugs over a long period of time, with high frequency of ADRs, which compromise the effectiveness of treatment. In addition, drug resistance has increased in recent years [10–12].

Following international recommendations, CD clinical management in Bolivia is supposed to be implemented in primary healthcare centers [13]. However, there was no model for care of adults with chronic infection, which led to the development of the first Chagas Platform in 2009 [14] by Barcelona Institute for Global Health (ISGlobal)–Fundació Clinic (Barcelona, Spain) and the Foundation for Applied Science and Studies for Health and Environmental Development (CEADES), promoted by the Spanish Agency for International Development Cooperation (AECID), aligned to the Chagas National Program (Bolivian Ministry of Health). Chagas Platforms are facilities that provide healthcare and expertise in CD management and building capacity for research; they train healthcare professionals in CD management, and promote educational activities in the community [14]. The project has grown and there are currently six Chagas Platforms working in Bolivia, four of them located in the department of Cochabamba. However, increasing access to CD care requires incorporating diagnosis and treatment into the national healthcare system. To this end, a network including Chagas Platforms, and primary and secondary healthcare centers was established in 2017 to strengthen (and, in the majority of health centers, to offer for the first time) diagnosis and treatment for CD [14]. Clinical management of TB in Bolivia takes place in primary and secondary healthcare centers, following international recommendations [15].

Poor adherence to treatment continues to be a challenge in the management of CD and TB. The main factors contributing to this are the lack of follow-up protocols during treatment, and drug toxicity. Nevertheless, with adequate drug management, most patients are able to finish treatment even with ADRs, which in CD are usually mild and well controlled with symptomatic treatment. Close medical follow-up, monitorization of ADRs and implementation of robust pharmacovigilance systems are essential factors for improving adherence and achieve therapeutic success [14, 16–19].

Recently, the importance of establishing strong and consolidated connections between public health programs and pharmacovigilance has been emphasized. Public health programs and pharmacovigilance are synergistic, since detecting and reporting ADRs will provide knowledge on how to improve patient safety and adherence to treatments [20].

In this context, strengthening the Bolivian pharmacovigilance system is proposed as a key issue for reinforcing therapeutic strategies for CD and TB. We hypothesized that implementing a strong and consolidated pharmacovigilance system would allow to better detect and report ADRs, and would provide knowledge on improving patient safety and adherence of treatments.

## Methodology

### Actors involved

The current project has been implemented by ISGlobal and CEADES. Both institutions developed the Bolivian Chagas Platform, a model for protocolized care of adults with *T*. *cruzi* infection, together with the Chagas National and Departmental Program; this increased the number of adults diagnosed and treated for CD [14]. The research was carried out in collaboration with the Pharmacovigilance Unit of the Bolivian Ministry of Health (UNIMED), the Pan American Health Organization (PAHO), the Departmental Chagas Program of Cochabamba (ChDP) and the Tuberculosis Departmental Program of Cochabamba (TBDP). A total of fifteen rural and urban primary and secondary healthcare centers participated in the study, including four healthcare centers of the Bolivian Chagas Platform.

### Intervention plan

**Fig 1:**
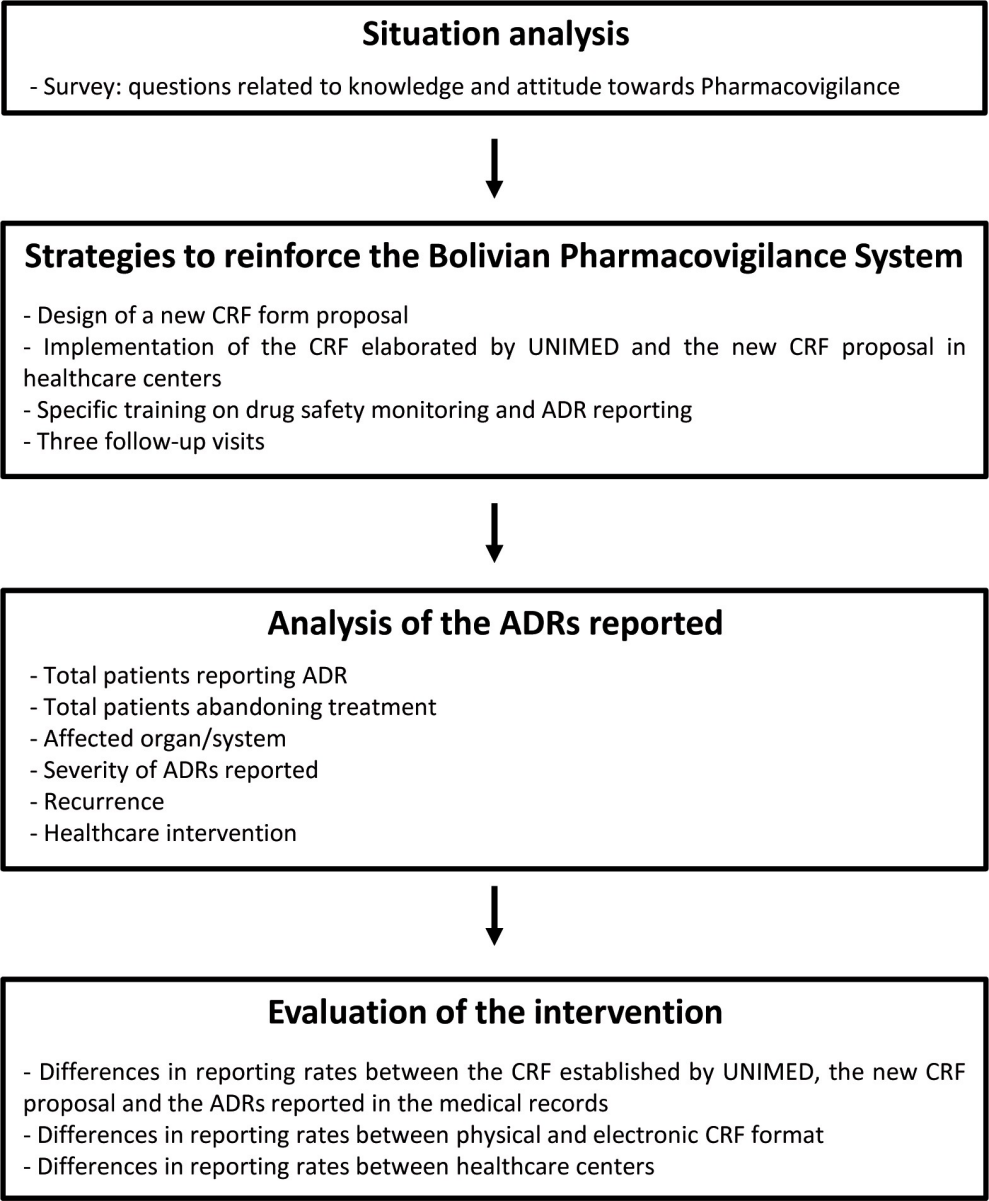
Flow chart summarizing the strategies followed during the intervention plan. A situation analysis survey was carried out to study in depth the causes of underreporting ADRs, followed by several strategies to reinforce Bolivian pharmacovigilance. At the end of the project, reported ADRs were analyzed and intervention was evaluated. CRF: case report form; UNIMED: Pharmacovigilance Unit of the Bolivian Ministry of Health; ADR: adverse drug reaction.

The intervention plan was performed in accordance with a flow chart (Fig 1). Detailed information concerning the intervention plan can be found in the supporting information material (S1 File).

### Data analysis

Absolute and relative frequency counts, and measures of central tendency (mean) were calculated. Adobe Photoshop 8.0 software was used for creating graphs.

### Ethics statement

The study protocol was approved by the Ethics Committee of the Hospital Clinic of Barcelona and the Ethics Committee of CEADES. Using anonymized databases, data was collected from medical records of patients starting treatment for CD or TB in the healthcare centers participating in the study. Informed consent was not required because the data was previously anonymized.

## Results

### Situation analysis: a challenging beginning

In our survey, we observed that healthcare professionals confirmed the importance of reporting ADRs, giving an average score of 9.6 out of 10. This figure contrasts with the effectively reported ADR rate: 4.7 out of 10.

**Fig 2:**
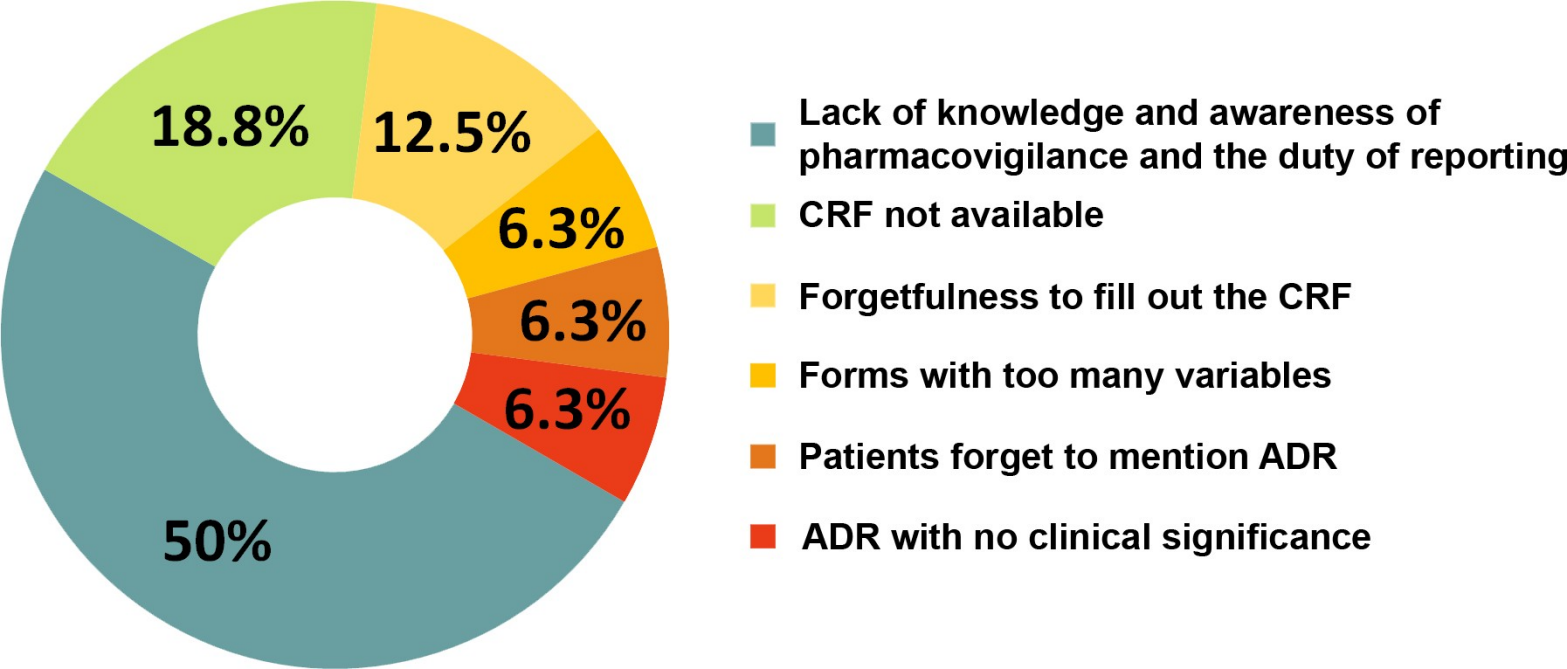
Causes for underreporting ADRs by healthcare professionals according to the results obtained from the situation analysis of pharmacovigilance in the Department of Cochabamba. **T**he most common (50%) reason for not reporting ADRs was "lack of knowledge and awareness of pharmacovigilance and the duty of reporting". CRF: case report form; ADR: adverse drug reaction.

Causes of underreporting ADRs by health professionals were multifaceted. The most common reason was "lack of knowledge and awareness of pharmacovigilance and the duty of reporting" (50%). Health professionals also reported "lack of availability of report forms" (18.8%), "forgetting to fill out the form" (12.5%), "forms with too many variables" (6.3%), "patients forgetting to mention the ADR" (6.3%) and "ADRs with no clinical significance" (6.3%) (Fig 2).

Out of all the healthcare professionals participating in the survey, 53% reported ADRs using patients’ medical records, 27% using case report forms (CRFs) established by UNIMED, and 20% using CRFs obtained from Bolivian ChDP. A total of 62.5% of healthcare professionals were not aware of the CRF established by UNIMED; of those who were, only 33,3% were able to fill out the form correctly.

Healthcare workers considered the most appropriate method to report ADRs was the use of electronic CRFs (50%), followed by the use of CRFs in physical format (31.3%); 18.8% had no preference.

Regarding reporting of ADRs to UNIMED, 43.8% of the healthcare professionals participating in our study considered all type of ADRs should be reported, 31.3% only moderate and severe ADRs, and 25% only severe ADRs.

Finally, a large percentage of healthcare professionals (81.3%) considered that receiving feedback from UNIMED would increase the reporting rate of ADRs.

### Evaluation of the intervention

**Fig 3:**
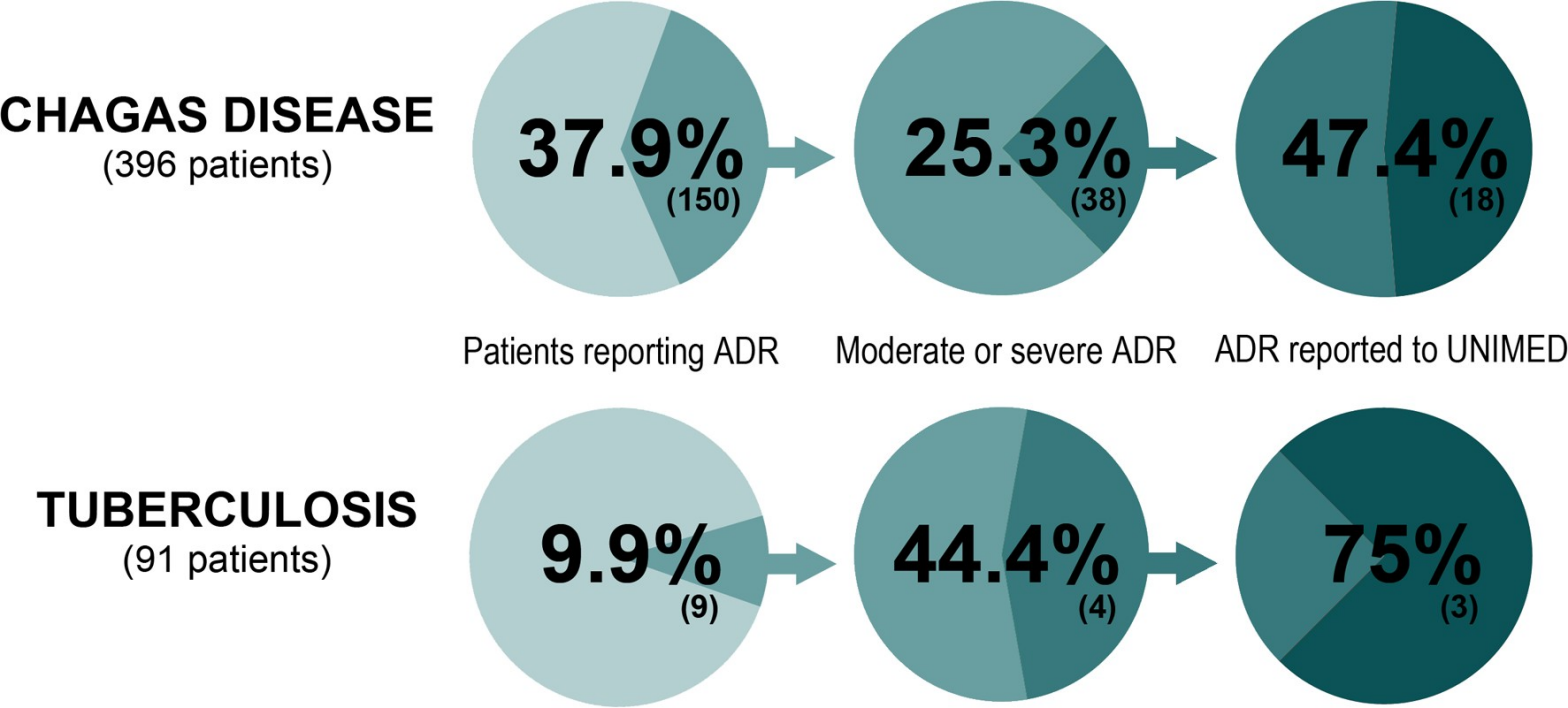
**Summary of the intervention results for CD (A) and TB (B). Total patients starting treatment for CD (A) or TB (B), reporting ADRs related to CD (A) or (TB) treatment according to the medical records, total ADRs classified as moderate or severe in the medical records, and total ADRs finally reported to the Bolivian pharmacovigilance system.** Less than half of all moderate and severe ADRs presented during CD treatment and collected in the medical records were reported to the Bolivian pharmacovigilance system. The figures are slightly better for TB, with 75% of the ADRs reported to UNIMED. ADR: adverse drug reaction; UNIMED: Pharmacovigilance Unit of the Bolivian Ministry of Health; CD: Chagas Disease; TB: Tuberculosis.

Using information contained in the medical records, we found that 37.9% of the 396 patients starting treatment for CD presented one or more ADRs. Out of all ADRs events collected in the medical records, 25.3% of them were classified as moderate or severe, and should have been reported to the Bolivian pharmacovigilance system. Only 47.4% of them were reported to UNIMED (Fig 3A).

Regarding the 91 patients starting TB treatment, 9.9% suffered ADRs. Out of all ADRs events collected in the medical records, 44% were classified as moderate or severe, and should have been reported to the Bolivian pharmacovigilance system; of these, 75% were reported to UNIMED (Fig 3B).

Interestingly, differences in reporting rates between the CRF established by UNIMED and the new proposed CRF were not found.

### Analysis of the ADRs reported

Out of the 396 patients participating in the study and starting treatment for CD, 150 (37.9%) reported one or more ADRs, and 57 abandoned treatment (14.4%). Out of the 91 patients starting treatment for TB, 9 (9.9%) reported one or more ADRs, and 2 abandoned treatment (2.2%) (Table 1).

**Table 1:** Total patients starting treatment, reporting ADRs, and abandoning treatment for CD and TB in the Bolivian Chagas Platforms and the primary and secondary national healthcare centers located in the Department of Cochabamba and included in the study. Data are shown by geographical area.

	Cercado	Sacaba	Punata	Villa Tunari	Total
**Chagas Disease**					
**Patients starting treatment**	198	47	59	92	396
**Patients reporting ADRs**	99 (50%)	22 (46.8%)	10 (17%)	19 (20.7%)	150 (37.9%)
**Patients abandoning treatment**	31 (15.7%)	10 (21.3%)	5 (8.5%)	11 (12%)	57 (14.4%)
**Tuberculosis**					
**Patients starting treatment**	45	27	5	14	91
**Patients reporting ADR**	7 (15.6%)	0 (0%)	1 (20%)	1 (7.1%)	9 (9.9%)
**Patients abandoning treatment**	2 (4.4%)	0 (0%)	0 (0%)	0 (0%)	2 (2.2%)

Concerning CD, 93 (35.4%) of the ADRs presented during treatment were dermatological, 76 (28.9%) affected the central nervous system, 52 (19.8%) were gastrointestinal, and 42 (16%) affected other organs or systems. Most of the ADRs presented during CD treatment were mild (112, representing 74.7% of the total), whereas 14.7% (22) were classified as mild/moderate, 10% (15) as moderate, and less than 1% (1) as severe. When focusing on the healthcare intervention, nearly all ADRs were treated in a primary healthcare center (148, representing 98.7%), and only 2 (1.3%) had to be referred. Finally, the majority of ADRs were non-recurrent (108, 72%), while 28% (42) were recurrent.

Regarding the characteristics of ADRs presented during TB treatment, 4 (36.4%) were dermatological, 4 (36.4%) gastrointestinal, and 3 (27.3%) affected the central nervous system. In terms of severity, 5 (55.6%) of the ADRs presented were classified as mild, 3 (33.3%) were considered moderate, and 1 (11.1%) was severe. Regarding healthcare intervention, 5 (55.6%) of the ADRs presented were treated in a primary healthcare center, 3 (33.3%) were referred, and 1 (11.1%) was not reported. Lastly, most of the ADRs presented were non-recurrent (7, 77.8%).

## Discussion

Pharmacovigilance is an important component of national healthcare systems. Data from pharmacovigilance systems are essential to ensure safety and effectiveness of drugs and to provide information concerning regulatory actions [21]. Although integration of public health programs with pharmacovigilance is crucial and highly encouraged by the World Health Organization [20], it is still an issue that needs to be addressed in Latin American countries. The efforts of the Bolivian Ministry of Health to increase access to treatment for CD and TB require strengthening the pharmacovigilance system.

Latin America is a culturally rich region of the world with diverse perspectives on health. Pharmacovigilance in this region suffers from the same shortcomings as it does in developed countries. Furthermore, it faces other difficulties, being the more relevant ones the inequitable health systems, high percentages of the population with no access to medical care, and the use of herbal home remedies not subject to control processes. Since the early 1990s, Latin American pharmacovigilance systems have developed considerably and are now making major efforts to report ADRs. However, these activities are recent and need reinforcement [22–24]. In the particular case of Bolivia, to our knowledge, this is the first time that a study of these characteristics has been performed. Similar studies have been carried out in other lower/middle income countries, where pharmacovigilance is still a relatively new concept [25–27]. All studies agree on the need for more research and literature, and for a stronger focus on the challenges and barriers to be overcome to promote and sustain pharmacovigilance in resource-limited settings, prioritizing country’s specific needs.

Focusing on the results of the situation analysis, the most common reason detected for not reporting ADRs was “lack of knowledge and awareness of pharmacovigilance and the duty of reporting”, followed far behind by “lack of availability of report forms” (50% and 18.8%, respectively). These results show that, in the specific context of Cochabamba, there was a serious lack of knowledge and awareness of pharmacovigilance. Given this, issues such as “lack of time” or “forgetting to fill out the form”, which could be relevant in more developed settings, take second place. The situation analysis results show clearly that, despite having tools to report ADRs, the Bolivian pharmacovigilance system was not well known to most health professionals. UNIMED, the ChDP and the TBDP together detected an extended lack of knowledge and awareness of pharmacovigilance and the duty of reporting, and underreporting ADRs in the forms provided by UNIMED. Insufficient training of healthcare professionals was the most important and urgent weakness detected, especially concerning the primary national healthcare centers located in rural areas. Training and follow-up on drug safety monitoring and ADR reporting was carried out for all health professionals involved in CD and TB treatment in the Department of Cochabamba. A more specific training on pharmacovigilance was provided to all health professionals working in the national health care centers of rural areas (departments of Villa Tunari and Punata).

Although our project has shown interesting results, it is important to highlight that reinforcing the Bolivian pharmacovigilance system requires persistence and an active follow-up with actors directly involved in the national health system. This study was developed as a one-year pilot project that emphasizes the importance of an educational intervention to change attitude towards ADR reporting among healthcare professionals. Its long-term effect cannot be evaluated yet, but continuous follow-up will be essential for tracking and improving the pharmacovigilance program’s success. Follow-up and evaluation of this specific intervention are currently led by UNIMED, which is the highest authority and is in charge of pharmacovigilance in the country. UNIMED is also continuing the training activities for all healthcare workers and implementing the strategies that were suggested at the end of the project.

Out of all the moderate and severe ADRs resulting from CD treatment and collected in the medical records, less than half were reported to the Bolivian pharmacovigilance system. Regarding TB, 25% of the moderate and severe ADRs collected in the medical records were not reported to the Bolivian pharmacovigilance system. Although these figures could be better, we believe it is a promising start, considering the results of the situational analysis at the beginning of the study; we consider that our intervention has improved the situation and that the strategies applied have been successful. However, using medical records as the gold standard for reporting ADRs is a major limitation: non-uniform and incomplete information regarding ADRs leads to inaccurate or unreported events to the pharmacovigilance regulatory agency.

It is important to note that healthcare professionals are more used to report ADRs related to TB than to CD to the Bolivian pharmacovigilance system, even though CD has been declared as a health priority for the Ministry of Health [28]. TBDP had a pioneering role in promoting the national ADR reporting system and providing training for healthcare professionals [29]. Due to the characteristics of the current therapeutic strategies and the scaling-up of Chagas treatment, ChDP should emphasize pharmacovigilance activities to help physicians in their clinical practice.

The rate of ADRs for CD and TB was 37.9% and 9.9%, respectively; and the percentage of patient abandonment was 14.4% in CD patients and 2.2% in TB patients. Thus, the rate of ADRs and early treatment interruption was low compared to previous studies performed in other countries [19, 30–31]. We have no explanation for this phenomenon, but we can speculate. One possible explanation is that close follow-up of patients during treatment and providing detailed information on the treatment characteristics, schedule and duration, might have a positive effect on adherence and low rate of ADRs. In this context, it is important to note that the close follow-up of patients that takes place in the Chagas Platforms usually cannot be carried out in primary healthcare centers due to the lack of human resources, and this may cause a bias in the rate of CD ADRs and early treatment interruption observed. In a similar vein, it is also important to highlight the role of nurses during TB treatment. Due to the characteristics of the Tuberculosis National Program of Bolivia, nurses perform an extremely close follow-up of the patients, which can include home-based nursing if necessary. Furthermore, although abandonment levels may not seem high in TB, TB treatment abandonment has fatal consequences for patients, which also become potential sources of infection and resistance to available drugs [32]. Patients withholding information to healthcare workers due to mistrust could be another possible explanation. Finally, another possible explanation could be the lack of knowledge of healthcare professionals when it comes to identifying ADRs. However, in our opinion this is the least plausible explanation. We detected a lack of consensus on the definition of ADRs in several medical records, in which symptoms probably associated with concomitant pathologies experienced by patients during CD or TB treatment were considered ADRs in several clinical records. Nevertheless, as previously mentioned, this study was not designed to explain this phenomenon and, therefore, we can only hypothesize.

Differences in reporting rates between the CRF established by UNIMED and the new proposed CRF were not found. One possible explanation is that healthcare professionals did not see the benefits of using the new tool. However, the new proposed CRF results in better data quality, by collecting information on the characteristics of ADRs in terms of severity, affected organ/system, clinical suspicion of recurrence, and healthcare intervention. The main differences between the CRF established by UNIMED and the new CRF proposal are summarized in Table 2.

**Table 2:** Summary of the main characteristics of the CRF established by UNIMED and the new proposed CRF. The new CRF form was based on the follow-up form used by the Bolivian Chagas Platforms, the CRF established by UNIMED, and CRFs used in other countries in the region. Increasing the information regarding the characteristics of ADRs was considered to be particularly important.

	UNIMED	New CRF proposal
**Patient data**	**✓✓**	**✓**
**Medical history**	**✓✓**	**✓✓**
**Purpose of drug prescription**	**✘**	**✓**
**Information related to the drug suspected of causing the ADR**	**✓✓**	**✓✓**
**Information related to concomitant drugs**	**✓✓**	**✓✓**
**Characteristics of the ADR**	**✓**	**✓✓✓**
**Healthcare professional data**	**✓**	**✓✓**
**Time required**	**✓✓**	**✓**
**Available online**	**✓ **	** ✓**

Better designed, user-friendly and standardized reporting forms would improve the capture of accurate information about ADRs [21]. New tools adapted to the reality of healthcare workers are needed in order to strengthen the current Bolivian ADR reporting system. Nevertheless, we believe this should not be the main priority in the context of pharmacovigilance in Bolivia. The implementation of new policies should prioritize strengthening pharmacovigilance activities and training: if continuous training is provided and healthcare professionals have a solid awareness of the importance of pharmacovigilance and the duty of reporting, they will notify ADRs, regardless of the CRF used.

Lack of internet access is still an important problem in some regions of Bolivia, especially in rural areas. In several rural and urban health care centers included in our study internet connectivity was limited, and it was not possible to implement the electronic CRF. Nevertheless, other possibilities could be explored in order to overcome this limitation. Apps or electronic devices working in offline mode could be an alternative [33]. Additionally, some of the healthcare facilities participating in the study are currently under renovation and internet access is being implemented, so it was considered important to have both forms (the CRF established by UNIMED and the new proposed CRF) available in electronic format in order to facilitate reporting.

Lack of knowledge by healthcare professionals regarding the classification of ADRs by severity was detected during the study. In several medical records, the mild/moderate category was found, which is not correct. As stated before, lack of consensus on definition of ADRs was also detected, which is also important for their clinical management. In our study, we considered an ADR to be any event classified as such in the medical records. There is a need for overcoming these limitations; even though strengthening the Bolivian pharmacovigilance system has proven to be a good strategy to improve patient health and increase adherence to CD and TB treatment, currently, the system still presents some weaknesses. All actors involved in our project suggested the implementation of the following policies: (i) strengthen pharmacovigilance activities in UNIMED, with external funding sources if necessary, at least in a preliminary phase; (ii) provide continued training in pharmacovigilance for healthcare professionals, validated by the Bolivian Ministry of Health and the PAHO; (iii) perform continuous surveillance in pharmacovigilance activities in healthcare centers through monitoring and evaluation programs provided by UNIMED (in a preliminary stage, this could require external technical support or funding sources); and (iv) integrate pharmacovigilance into the curricula of medicine, pharmacy and nursing schools, to improve training of future healthcare professionals.

Overall, our findings suggest that the Bolivian pharmacovigilance system still presents some challenges that should be addressed in the coming years in order to achieve a strong, integrated and consolidated ADR reporting system. The reinforcement of the Bolivian pharmacovigilance system is an ambitious project that should involve a long-term perspective with several steps to follow. Our work was mainly focused on the analysis of ADR management and reporting, in primary healthcare level and at other levels. However, in order to improve the pharmacovigilance system, all stakeholders at different levels must work within their roles and responsibilities. UNIMED's current approach is to strengthen the Bolivian pharmacovigilance system in all care levels, focusing on other neglected and prevalent pathologies in Bolivia and including all the departments of the country. Several of our proposals are already in the process of being implemented. However, the responsibility of leading these actions is still unclear. Further work is needed in order to achieve a strong and consolidated ADR reporting system to improve patient safety, drug efficacy and adherence to treatment. A medium and long-term follow up to evaluate the impact of these actions is also required.

## Supporting information

S1 File Detailed methodology and strategies developed through the intervention plan. (DOCX)

S2 File Situation analysis survey. (DOCX)

S3 File Situation analysis survey results database. (DOCX)

S4 File Summary of the main relevant data (total patients starting treatment, completing treatment, abandoning treatment, total ADRs reported in medical reports, and total ADRs reported to UNIMED), and detailed information about the ADRs collected in the clinical records by healthcare facility. (XLSX)
