# Supplementary material for: Strengthening the Bolivian pharmacovigilance system: New surveillance strategies to improve care for Chagas disease and tuberculosis
Source: PLoS Negl Trop Dis. 2020 Sep 21;14(9):e0008370. doi: 10.1371/journal.pntd.0008370 (PMC7529217; doi:10.1371/journal.pntd.0008370)
Supplement: S1 File — (DOCX) [file pntd.0008370.s001.docx]

**Situation analysis**

A situation analysis of Pharmacovigilance in the Department of Cochabamba was performed in order to study in depth the causes of underreporting adverse drug reactions (ADRs). We collected data using a survey which included questions related to knowledge and attitude towards Pharmacovigilance (S2 File). All health professionals involved in Chagas disease (CD) treatment with at least one year of experience working in the national health system were selected to participate. After carrying out the situation analysis, the Pharmacovigilance Unit of the Bolivian Ministry of Health (UNIMED) proposed including tuberculosis (TB) in the project.

**Strategies to reinforce the current Bolivian Pharmacovigilance System**

Strengthening the Bolivian Pharmacovigilance system was proposed in our project as a key issue to reinforce therapeutic strategies for CD and TB. The Bolivian Pharmacovigilance system is based on healthcare professionals filling out the local case report form (CRF) developed by UNIMED. This form is sent to the healthcare network management, and it then arrives to UNIMED. Together with local health entities and UNIMED, the implementation of a new CRF was suggested to the Bolivian Ministry of Health. The new form was designed by a multidisciplinary team including healthcare workers, researchers, and health policymakers, based on the follow-up form used in the Bolivian Chagas Platforms, the CRF established in the national health system by UNIMED, and CRFs used in other countries of the region, similar to Bolivia but with a more solid experience in Pharmacovigilance. The objective was to develop a better designed, user-friendly and practical tool to provide good quality information, following the guidelines on good pharmacovigilance practices. Increasing the information regarding the characteristics of ADRs was considered to be particularly important.

The CRF elaborated by UNIMED and the new CRF proposal were implemented in the Bolivian Chagas Platforms located in the Department of Cochabamba (two in an urban area and two in rural areas), and in the primary and secondary national healthcare centers in the network. The CRF provided by UNIMED was already established in some of the healthcare facilities. Both forms were available in physical and electronic format, which was assigned randomly to each healthcare center. To evaluate the intervention, a comparative analysis of ADR reporting was carried out using the CRF established by UNIMED, the new proposed CRF, and the medical records. The medical records of all patients starting treatment for CD or TB were analyzed in order to detect how many of them experienced ADRs during treatment.

Together with the Pan American Health Organization (PAHO) and UNIMED, a specific training on drug safety monitoring and ADR reporting was given to all health professionals involved in CD and TB treatment. Three follow-up visits were performed in each healthcare center.

**Analysis of the ADRs reported**

We analyzed the ADRs reported, focusing on the following variables: total patients reporting ADR; total patients abandoning treatment; affected organ/system; severity of ADRs; recurrence of ADRs; healthcare intervention; differences in reporting rates between the CRF established by UNIMED, the new CRF proposal and the ADRs reported in the medical records; differences in reporting rates between physical and electronic CRF format; and differences in reporting rates between healthcare centers.

Patient abandonment was defined as failure to complete the medically indicated curative therapy.
