# Supplementary material for: Strengthening the Bolivian pharmacovigilance system: New surveillance strategies to improve care for Chagas disease and tuberculosis
Source: PLoS Negl Trop Dis. 2020 Sep 21;14(9):e0008370. doi: 10.1371/journal.pntd.0008370 (PMC7529217; doi:10.1371/journal.pntd.0008370)
Supplement: S2 File — (DOCX) [file pntd.0008370.s002.docx]

# 1 S2 File. Situation analysis survey.

2

# 1. On a scale of 1 (never) to 7 (always), indicate the degree of frequency in which you report

1. **the unwanted drug-related adverse events (ADRs).**

5 1 2 3 4 5 6 7

# 2. Indicate the reasons for not reporting ADRs.

1. (Open answer. If there is no response, give the following options)
2. Lack of knowledge and awareness of pharmacovigilance and the duty of reporting.
3. Lack of knowledge and awareness of how to report ADRs.
4. Lack of knowledge and awareness of when to report ADRs.
5. Time pressure.
6. Lack of availability of CRF forms in the health centers.
7. Unclear forms.
8. Forms presenting too much information.
9. Patient communication problems.
10. Reporting ADRs is useless and time consuming.
11. Other reasons: _

# 3. In your opinion, which would be the most appropriate method to report ADRs?

1. (Open answer. If there is no response, give the following options)
2. CRFs in physical format.
3. Electronic CRFs.
4. By phone.

# 4. In your opinion, which ADRs should be reported to UNIMED?

# (Open answer. If there is no response, give the following options)

# All of them.

1. Severe and moderate.
2. Mild.
3. None.

# 5. Are you aware of the CRF established by UNIMED?

1. Yes No

# 6. In your opinion, when should the CRF be filled in?

1. (Open answer. If there is no response, give the following options)
2. When the ADR is detected.
3. Once the ADR has remitted.
4. It is necessary to follow up on the ADR, starting once the ADR is detected and finishing when
5. the patient is fully recovered.

# 7. Would you report ADRs more frequently if you received feedback about the data

1. **provided?**
2. Yes No
3. **8. On a scale of 1 (not important at all) to 7 (very important), in your opinion, what is**
4. **the importance of reporting ADRs**?

1 2 3 4 5 6 7
