# Supplementary material for: Strengthening the Bolivian pharmacovigilance system: New surveillance strategies to improve care for Chagas disease and tuberculosis
Source: PLoS Negl Trop Dis. 2020 Sep 21;14(9):e0008370. doi: 10.1371/journal.pntd.0008370 (PMC7529217; doi:10.1371/journal.pntd.0008370)
Supplement: S3 File — (DOCX) [file pntd.0008370.s003.docx]

**S2 File. Situation analysis survey results.**

**PLATAFORMA SACABA (1)**

**1. On a scale of 1 (never) to 7 (always), indicate the degree of frequency in which you report the unwanted drug-related adverse events (ADRs).**

1 2 3 4 5 6 7

**2. Indicate the reasons for not reporting ADRs.**

Health care professionals being lazy, forgetfulness of reporting ADR (it takes considerable time from seeing the patient until reporting ADR).

**3. In your opinion, which would be the most appropriate method to report ADRs?**

Online.

**4. In your opinion, which ADRs should be reported to UNIMED?**

When the suffered ADR implies suspending the treatment, it is necessary to report to UNIMED. If all ADRs had to be reported, everything would be reported.

**5. Are you aware of the CRF established by UNIMED?**

Yes.

**6. In your opinion, when should the CRF be filled in?**

Once the patient is OK and the ADR has remitted.

**7. Would you report ADRs more frequently if you received feedback about the data provided?**

Yes.

**8. On a scale of 1 (not important at all) to 7 (very important), in your opinion, what is the importance of reporting ADRs?**

1 2 3 4 5 6 7

**Other comments:** It is important to check if the drug is useful, and to look for other options if it is necessary. It is also important to check the drug batch number.

**PLATAFORMA CERCADO (1)**

**1. On a scale of 1 (never) to 7 (always), indicate the degree of frequency in which you report the unwanted drug-related adverse events (ADRs).**

1 2 3 4 5 6 7

**2. Indicate the reasons for not reporting ADRs.**

Patients forgetting to report ADRs: patients forget to report ADRs to the medical doctor, and once the medical doctor meets the patient the ADR is over.

Lack of time.

**3. In your opinion, which would be the most appropriate method to report ADRs?**

Online.

**4. In your opinion, which ADRs should be reported to UNIMED?**

Severe ADRs.

**5. Are you aware of the CRF established by UNIMED?**

Yes.

**6. In your opinion, when should the CRF be filled in?**

Once the ADR is severe, immediately, as soon as the ADR is detected in the patient.

**7. Would you report ADRs more frequently if you received feedback about the data provided?**

Yes.

**8. On a scale of 1 (not important at all) to 7 (very important), in your opinion, what is the importance of reporting ADRs?**

1 2 3 4 5 6 7

**PLATAFORMA CERCADO (2)**

**1. On a scale of 1 (never) to 7 (always), indicate the degree of frequency in which you report the unwanted drug-related adverse events (ADRs).**

1 2 3 4 5 6 7

**2. Indicate the reasons for not reporting ADRs.**

Forms get lost.

**3. In your opinion, which would be the most appropriate method to report ADRs?**

Online because it is faster.

In physical format for not losing information online.

**4. In your opinion, which ADRs should be reported to UNIMED?**

Moderate and severe ADRs, but we never see that type of ADRs in our health center.

Mild ADRs are reported in the monthly report.

**5. Are you aware of the CRF established by UNIMED?**

Yes.

**6. In your opinion, when should the CRF be filled in?**

We should treat the patient and immediately report.

**7. Would you report ADRs more frequently if you received feedback about the data provided?**

Yes.

**On a scale of 1 (not important at all) to 7 (very important), in your opinion, what is the importance of reporting ADRs?**

1 2 3 4 5 6 7

**PLATAFORMA CERCADO (3)**

**1. On a scale of 1 (never) to 7 (always), indicate the degree of frequency in which you report the unwanted drug-related adverse events (ADRs).**

1 2 3 4 5 6 7

**2. Indicate the reasons for not reporting ADRs.**

There are no instructions on notifying UNIMED.

We report ADRs using a monthly report.

**3. In your opinion, which would be the most appropriate method to report ADRs?**

Online.

Physical forms take time and get lost.

**4. In your opinion, which ADRs should be reported to UNIMED?**

In the monthly report we report all ADRs, including the mild ones. In the Plataformas we do not see severe ADRs, but we see moderate ones. Moderate ADRs are included in the monthly report.

**5. Are you aware of the CRF established by UNIMED?**

I have never filled in the CRF established by UNIMED.

**6. In your opinion, when should the CRF be filled in?**

I have never filled in the CRF established by UNIMED.

**7. Would you report ADRs more frequently if you received feedback about the data provided?**

No.

**8. On a scale of 1 (not important at all) to 7 (very important), in your opinion, what is the importance of reporting ADRs?**

1 2 3 4 5 6 7

**Other comments:** Nobody notifies ADRs, not even the patients. People are not used to report (not the doctors and neither the patients).

**PLATAFORMA CERCADO (4)**

**1. On a scale of 1 (never) to 7 (always), indicate the degree of frequency in which you report the unwanted drug-related adverse events (ADRs).**

1 2 3 4 5 6 7

**2. Indicate the reasons for not reporting ADRs.**

Lack of causality between the drug and the ADR.

Mild ADRs without clinical relevance.

**3. In your opinion, which would be the most appropriate method to report ADRs?**

It is irrelevant to have the form in physical or electronic format.

To have the information presented as a form facilitates the reporting process.

**4. In your opinion, which ADRs should be reported to UNIMED?**

In the patient’s medical record, all ADRs.

Those ADRs requiring hospitalization (moderate and severe) must be reported to the Ministry of Health.

**5. Are you aware of the CRF established by UNIMED?**

Yes.

**6. In your opinion, when should the CRF be filled in?**

As soon as the ADR is detected in the patient.

The form must be kept together with the patient’s clinical record. As the patient is followed up, the form is filled in.

**7. Would you report ADRs more frequently if you received feedback about the data provided?**

Yes.

**8. On a scale of 1 (not important at all) to 7 (very important), in your opinion, what is the importance of reporting ADRs?**

1 2 3 4 5 6 7

**PLATAFORMA PUNATA (1)**

**1. On a scale of 1 (never) to 7 (always), indicate the degree of frequency in which you report the unwanted drug-related adverse events (ADRs).**

1 2 3 4 5 6 7

**Other comments:** I report moderate and severe ADRs, but not mild ones.

Moderate and severe ADRs are not frequently seen in Plataformas.

**2. Indicate the reasons for not reporting ADRs.**

Lack of knowledge. Until last year I was not aware of the form provided by UNIMED and the duty of reporting.

Also, reporting ADRs appears to be time consuming.

**3. In your opinion, which would be the most appropriate method to report ADRs?**

Online.

Nowadays, UNIMED forms are kept together with patient’s clinical records, and this is not useful. No one from UNIMED gets the forms, and the forms get lost, nothing is done with the information.

**4. In your opinion, which ADRs should be reported to UNIMED?**

Severe and moderate.

**5. Are you aware of the CRF established by UNIMED?**

Yes. After filling out the form, this is kept together with patient’s medical record.

**6. In your opinion, when should the CRF be filled in?**

Once the ADR is detected.

**7. Would you report ADRs more frequently if you received feedback about the data provided?**

Yes.

**8. On a scale of 1 (not important at all) to 7 (very important), in your opinion, what is the importance of reporting ADRs?**

1 2 3 4 5 6 7

**CHIPIRIRI (1)**

**1. On a scale of 1 (never) to 7 (always), indicate the degree of frequency in which you report the unwanted drug-related adverse events (ADRs).**

1 2 3 4 5 6 7

**2. Indicate the reasons for not reporting ADRs.**

Forms presenting too many variables.

**3. In your opinion, which would be the most appropriate method to report ADRs?**

CRFs in physical format.

Internet stops working frequently.

**4. In your opinion, which ADRs should be reported to UNIMED?**

All of them.

**5. Are you aware of the CRF established by UNIMED?**

No. We report ADRs using patient’s medical records.

**6. In your opinion, when should the CRF be filled in?**

I am not aware of the CRF established by UNIMED.

**7. Would you report ADRs more frequently if you received feedback about the data provided?**

Yes.

**8. On a scale of 1 (not important at all) to 7 (very important), in your opinion, what is the importance of reporting ADRs?**

1 2 3 4 5 6 7

**PLATAFORMA VILLA TUNARI (1)**

**1. On a scale of 1 (never) to 7 (always), indicate the degree of frequency in which you report the unwanted drug-related adverse events (ADRs).**

1 2 3 4 5 6 7

**2. Indicate the reasons for not reporting ADRs.**

There is an important lack of Pharmacovigilance in Bolivia: Pharmacovigilance is not well known.

We have never received training on Pharmacovigilance or on the UNIMED reporting form.

**3. In your opinion, which would be the most appropriate method to report ADRs?**

CRF forms in physical format to notify weekly.

Frequently, internet connection doesn’t work (due to the rain…).

**4. In your opinion, which ADRs should be reported to UNIMED?**

All of them.

**5. Are you aware of the CRF established by UNIMED?**

I’m not aware of the UNIMED reporting form.

We report mild, moderate and severe ADRs using the specific F3 form of the Plataforma. We report to management (SEDES) monthly.

**6. In your opinion, when should the CRF be filled in?**

I’m not aware of the UNIMED reporting form.

**7. Would you report ADRs more frequently if you received feedback about the data provided?**

Yes.

**8. On a scale of 1 (not important at all) to 7 (very important), in your opinion, what is the importance of reporting ADRs?**

1 2 3 4 5 6 7

**SAN GABRIEL (1)**

**1. On a scale of 1 (never) to 7 (always), indicate the degree of frequency in which you report the unwanted drug-related adverse events (ADRs).**

1 2 3 4 5 6 7

**2. Indicate the reasons for not reporting ADRs.**

Lack of forms availability (specifically in Villatunari, no one has this material).

Lack of knowledge about Pharmacovigilance and the available resources.

Lack of knowledge and awareness about the duty of reporting ADRs.

Lack of time.

**3. In your opinion, which would be the most appropriate method to report ADRs?**

CRFs forms in electronic format.

**4. In your opinion, which ADRs should be reported to UNIMED?**

Severe. However, I have never seen severe ADRs with benznidazole treatment.

**5. Are you aware of the CRF established by UNIMED?**

I’m not aware of the UNIMED reporting form, I have never seen it before.

We report weekly using patient’s clinical records.

**6. In your opinion, when should the CRF be filled in?**

I’m not aware of the UNIMED reporting form.

**7. Would you report ADRs more frequently if you received feedback about the data provided?**

Yes.

**8. On a scale of 1 (not important at all) to 7 (very important), in your opinion, what is the importance of reporting ADRs?**

1 2 3 4 5 6 7

**TICTI NORTE (1)**

**1. On a scale of 1 (never) to 7 (always), indicate the degree of frequency in which you report the unwanted drug-related adverse events (ADRs).**

1 2 3 4 5 6 7

**2. Indicate the reasons for not reporting ADRs.**

Forgetfulness of filling out the forms and report to the program.

**3. In your opinion, which would be the most appropriate method to report ADRs?**

It is irrelevant if the CRF is available in physical or electronic format.

It would be important to have a person in charge of reporting for each program.

**4. In your opinion, which ADRs should be reported to UNIMED?**

All of them.

**5. Are you aware of the CRF established by UNIMED?**

I’m not aware of the UNIMED reporting form.

Currently we report using patient’s clinical records.

**6. In your opinion, when should the CRF be filled in?**

I’m not aware of the UNIMED reporting form.

**7. Would you report ADRs more frequently if you received feedback about the data provided?**

Yes.

**8. On a scale of 1 (not important at all) to 7 (very important), in your opinion, what is the importance of reporting ADRs?**

1 2 3 4 5 6 7

**SARCOBAMBA (1)**

**1. On a scale of 1 (never) to 7 (always), indicate the degree of frequency in which you report the unwanted drug-related adverse events (ADRs).**

1 2 3 4 5 6 7

**2. Indicate the reasons for not reporting ADRs.**

Lack of knowledge about the duty of reporting and the protocols to be followed.

Personal criteria of the medical doctor.

**3. In your opinion, which would be the most appropriate method to report ADRs?**

Forms in physical format. Electronic CRFs would be ideal, but there is not internet in the health center.

**4. In your opinion, which ADRs should be reported to UNIMED?**

ADRs that can be not handled in the health center, and need to be referred.

**5. Are you aware of the CRF established by UNIMED?**

I’m not aware of the UNIMED reporting form.

We use a CRF provided by the Chagas program initiated by MSF. This form is filled in once the ADR is detected in the patient, and it is reported to management of Cercado network once a month.

**6. In your opinion, when should the CRF be filled in?**

I’m not aware of the UNIMED reporting form.

**7. Would you report ADRs more frequently if you received feedback about the data provided?**

No. I think I would report more ADRs if specific training was provided to the health care workers.

**8. On a scale of 1 (not important at all) to 7 (very important), in your opinion, what is the importance of reporting ADRs?**

1 2 3 4 5 6 7

**SEBASTIAN PAGADOR (1)**

**1. On a scale of 1 (never) to 7 (always), indicate the degree of frequency in which you report the unwanted drug-related adverse events (ADRs).**

1 2 3 4 5 6 7

**2. Indicate the reasons for not reporting ADRs.**

We are not aware of the forms or the duty of reporting ADRs.

We have never received training or information on Pharmacovigilance.

**3. In your opinion, which would be the most appropriate method to report ADRs?**

Forms in physical format, even though they can get lost. Electronic CRFs would be ideal, but there is not internet in our health center.

**4. In your opinion, which ADRs should be reported to UNIMED?**

All of them.

**5. Are you aware of the CRF established by UNIMED?**

I’m not aware of the UNIMED reporting form.

We report ADRs using patient’s clinical records.

**6. In your opinion, when should the CRF be filled in?**

I’m not aware of the UNIMED reporting form.

**7. Would you report ADRs more frequently if you received feedback about the data provided?**

Yes.

**8. On a scale of 1 (not important at all) to 7 (very important), in your opinion, what is the importance of reporting ADRs?**

1 2 3 4 5 6 7

**PUCARITA (1)**

**1. On a scale of 1 (never) to 7 (always), indicate the degree of frequency in which you report the unwanted drug-related adverse events (ADRs).**

1 2 3 4 5 6 7

**2. Indicate the reasons for not reporting ADRs.**

Lack of knowledge about the reporting forms.

We do not report ADRs since MSF stopped working with us. MSF used to provide all the forms.

**3. In your opinion, which would be the most appropriate method to report ADRs?**

Forms in physical format.

CRFs should be user-friendly.

**4. In your opinion, which ADRs should be reported to UNIMED?**

All of them.

**5. Are you aware of the CRF established by UNIMED?**

I’m not aware of the UNIMED reporting form.

We report ADRs internally using patient’s medical records.

**6. In your opinion, when should the CRF be filled in?**

I’m not aware of the UNIMED reporting form.

**7. Would you report ADRs more frequently if you received feedback about the data provided?**

No. We consider that the most important point of Pharmacovigilance is to provide accurate information to the patients about possible ADRs that they can suffer.

**8. On a scale of 1 (not important at all) to 7 (very important), in your opinion, what is the importance of reporting ADRs?**

1 2 3 4 5 6 7

T**ARATA (1)**

**1. On a scale of 1 (never) to 7 (always), indicate the degree of frequency in which you report the unwanted drug-related adverse events (ADRs).**

1 2 3 4 5 6 7

**2. Indicate the reasons for not reporting ADRs.**

Report forms not available in our health center.

**3. In your opinion, which would be the most appropriate method to report ADRs?**

Online.

**4. In your opinion, which ADRs should be reported to UNIMED?**

Severe.

**5. Are you aware of the CRF established by UNIMED?**

We report ADRs using specific CRFs provided by the Departmental Chagas Program.

I’m not aware of the UNIMED reporting form.

**6. In your opinion, when should the CRF be filled in?**

I’m not aware of the UNIMED reporting form.

**7. Would you report ADRs more frequently if you received feedback about the data provided?**

Yes.

**8. On a scale of 1 (not important at all) to 7 (very important), in your opinion, what is the importance of reporting ADRs?**

1 2 3 4 5 6 7

**TOLATA (1)**

**1. On a scale of 1 (never) to 7 (always), indicate the degree of frequency in which you report the unwanted drug-related adverse events (ADRs).**

1 2 3 4 5 6 7

**2. Indicate the reasons for not reporting ADRs.**

Lack of knowledge about the obligation of reporting ADRs.

**3. In your opinion, which would be the most appropriate method to report ADRs?**

Online user-friendly forms.

**4. In your opinion, which ADRs should be reported to UNIMED?**

All of them.

**5. Are you aware of the CRF established by UNIMED?**

We report ADRs using specific CRFs provided by the Departmental Chagas Program.

I’m not aware of the UNIMED reporting form.

**6. In your opinion, when should the CRF be filled in?**

I’m not aware of the UNIMED reporting form.

**7. Would you report ADRs more frequently if you received feedback about the data provided?**

Yes.

**8. On a scale of 1 (not important at all) to 7 (very important), in your opinion, what is the importance of reporting ADRs?**

1 2 3 4 5 6 7

**HOSPITAL MEXICO (1)**

**1. On a scale of 1 (never) to 7 (always), indicate the degree of frequency in which you report the unwanted drug-related adverse events (ADRs).**

1 2 3 4 5 6 7

**2. Indicate the reasons for not reporting ADRs.**

-

**3. In your opinion, which would be the most appropriate method to report ADRs?**

Current reporting forms in physical format.

Internet is not available in this health center.

**4. In your opinion, which ADRs should be reported to UNIMED?**

All of them.

**5. Are you aware of the CRF established by UNIMED?**

Yes.

**6. In your opinion, when should the CRF be filled in?**

Once ADRs are detected.

**7. Would you report ADRs more frequently if you received feedback about the data provided?**

Yes.

**8. On a scale of 1 (not important at all) to 7 (very important), in your opinion, what is the importance of reporting ADRs?**

1 2 3 4 5 6 7
